# Highlights From the Successful Aging After Elective Surgery (SAGES) I Study: Fifteen Years of Findings

**DOI:** 10.1093/geroni/igaf122.1051

**Published:** 2025-12-31

**Authors:** Sarinnapha Vasunilashorn

## Abstract

Delirium, an acute change in cognition, is common, morbid, and costly geriatric syndrome. Although the epidemiology of delirium has been well-characterized, the relationship of delirium with long-term cognitive and functional outcomes remains unclear. Moreover, less is known of the biological underpinnings of postoperative delirium and its associated long-term outcomes. Funded by an NIA program project, the five-year prospective Successful Aging after Elective Surgery (SAGES) I study was launched in 2010 to investigate novel risk factors (e.g., biomarkers, neuroimaging, reserve markers) and to examine the contribution of delirium to long-term cognitive and functional decline. The SAGES I study enrolled 560 adults age ≥70 undergoing major noncardiac surgery who did not have evidence of dementia based on detailed screening process (including a complete baseline neuropsychological test battery and a functional status battery). Fifteen years following enrollment of the first SAGES I participant, this symposium highlights the select key findings of the SAGES I study, including the: 1) relationship between functional recovery following postoperative delirium, 2) association between postoperative delirium and long-term cognitive decline 72 months post-surgery, 3) the association between a novel marker of neural injury, phosphorylated-tau 217, and delirium, and 4) identification of preoperative and postoperative blood biomarkers associated with delirium using a proteomics approach. Taken together, these studies summarize gaps in knowledge that the SAGES study has addressed and paves the way for future promising lines of inquiry in delirium research-expanding our understanding of this geriatric syndrome that threatens the independence and quality of life of older adults.

